# 

**Published:** 1896-10

**Authors:** 


### CONTENTS.

ORIGINAL ARTICLES:
Social Position of the Veterinarian. By J. W. SALLADE, V.S. . . . . . 717
Abstract of a Paper on Veterinary Dentistry. By R. C. HURLBURT, D.V.S. . 720
ABSTRACTS FROM FOREIGN JOURNALS:
Septicæmia Hæmorrhagica—Ovarian Dropsy in a Cow . . , . . . 722–725
REPORTS OF CASES:
Actinomycosis of the Inferior Maxilla in a Horse. By L. E. WILLYOUNG, D.V.S. 726
Fistulous Withers Treated by Actual Cautery. By JAMES A. WAUGH, V.S. . 728
CONTROL WORK:
Massachusetts — Connecticut — New York — Pennsylvania—Virginia—Alabama—Indiana—Missouri—Iowa—California—South Africa . . . . . 730–733
EDITORIALS:
The 1897 Meeting-place of the U. S. V. M. A.—Dangers of National Associations—Massachusetts Cattle Commission . . . . . . . . 734–735
NECROLOGY—Dr. W. D. Southwick—Dr. Charles S. Douglass . . . . . 737
AFTERMATH . . . . . . . . . . . . . . . . . 738
FAR AND NEAR . . . . . . . . . . . . . . . 739
PERSONAL . . . . . . . . . . . . . . . . . 741
SOCIETY PROCEEDINGS:
Schuylkill Valley Veterinary Medical Association—New Hampshire Veterinary Medical Association—Association of Veterinary Faculties of North America—New York State Veterinary Medical Association—Associations and Association Meetings—U. S. V. M. A. Appointments . . . . . . . . 744–750
AMONG THE COLLEGES:
Veterinary Department, Ohio State University—Chicago Veterinary College—National Veterinary College—McGill University . . . . . . 750–751
GLEANINGS . . . . . . . . . . . . . . . . . 751

